# Morphological Evaluation of Supramolecular Soft Materials Obtained through Co-Assembly Processes

**DOI:** 10.3390/gels9110886

**Published:** 2023-11-09

**Authors:** Alexandra Croitoriu, Aurica P. Chiriac, Alina G. Rusu, Alina Ghilan, Diana E. Ciolacu, Iuliana Stoica, Loredana E. Nita

**Affiliations:** “Petru Poni” Institute of Macromolecular Chemistry, 41-A Grigore Ghica Voda Alley, 700487 Iasi, Romania; croitoriu.alexandra@icmpp.ro (A.C.); achiriac@icmpp.ro (A.P.C.); diaconu.alina@icmpp.ro (A.G.); dciolacu@icmpp.ro (D.E.C.); stoica_iuliana@icmpp.ro (I.S.)

**Keywords:** low-molecular-weight gelators, amino acids, gel, self-assembly

## Abstract

Low-molecular-weight gelators (LMWGs) are compounds with an intrinsic tendency to self-assemble forming various supramolecular architectures via non-covalent interactions. Considering that the development of supramolecular assemblies through the synergy of molecules is not entirely understood at the molecular level, this study introduced a Fmoc-short peptide and four Fmoc-amino acids as building blocks for the self-assembly/co-assembly process. Hence, we investigated the formation of supramolecular gels starting from the molecular aggregation following two triggering approaches: solvent/co-solvent method and pH switch. The complex morphological analysis (POM, AFM, and STEM) offered an insight into the spontaneous formation of well-ordered nanoaggregates. Briefly, POM and AFM images demonstrated that self-assembled gels present various morphologies like dendrimer, spherulite, and vesicle, whereas all co-assembled supramolecular systems exhibit fibrillar morphologies as a result of the interaction between co-partners of each system. STEM study has confirmed that the molecules interact and join together, finally forming a fibrous network, an aspect seen in both self-assembled and co-assembled gels. XRD allowed the determination of the molecular arrangement. The study emphasized that the Fmoc motif protected the amino groups and facilitated gelation through additional π-π interactions.

## 1. Introduction

Supramolecular soft materials formed by the self-assembly of low-molecular-weight gelators (LMWGs) have attracted considerable attention in many fields because of their impressive inherent features [1]. The potential of supramolecular soft materials is being widely explored as an excellent platform for bioapplications (tissue engineering, drug delivery, therapeutics, biosensing, biomonitoring, etc.). In addition, due to their special features including solid-like rheology and swelling–shrinking behaviors, gels and hydrogels have found applicability in many areas including shape memory engineering [2], actuators [3] and ophthalmological viscoelastic devices [4]. The versatile character of these materials is given by modern gel preparation techniques that pursue a cost-effective, solvent-free, and energy-efficient technique [2]. 

Under specific conditions, LMWGs exhibit a “bottom-up” [5] spontaneous self-organization. In brief, the molecules are organized into a one-dimensional anisotropic nanoaggregate that subsequently adopts a fairly stable 3D network. The advantage of using amino acids or peptides as LMWGs emerges from the fact that, in their composition, two functional domains are simultaneously involved, the hydrophilic region that favors the interaction with water molecules, and the hydrophobic region that indicates potential in the formation of Van der Waals forces or hydrophobic interactions. Short peptides and amino acids (AA) protected at the N-terminal with a synthetic aromatic motif as fluorenyl methoxycarbonyl (Fmoc) are amphiphilic compounds used as LMWGs because of their hydrophilic-lipophilic balance and chemical complementarity [6]. The short peptide/AA self-assembly process is influenced by intrinsic (the sequence of peptide, the nature of the amino acid side chains, hydrophilicity, and hydrophobicity) and external factors (pH switch, organic solvent assistance, temperature, ionic strength adjustments, and sonication). By adjusting these factors, the Fmoc-modified peptide/AA adopts different architectures such as nanofibrils [7], nanotubes [8], and nanoaggregates. The driving forces behind the self-assembly process include a tremendous variety of non-covalent interactions generated by the amino acid side chains: π-π stacking (aromatic amino acids), hydrophobic interactions (nonpolar amino acids), hydrogen bonds, and electrostatic interactions (charged and uncharged polar amino acids) [9]. 

Several Fmoc-amino acids or peptides can self-assemble into supramolecular gels via non-covalent forces. For example, Gazit et al. [10] developed gels by dissolving Fmoc-Lys(Fmoc)-Arg-Gly-Asp [Fmoc-K(Fmoc)-RGD] in a miscible water–solvent mixture, whereas Gröhn et al. [11] reported a supramolecular architecture transition triggered by light irradiation. In addition, Adams et al. [12] used a pH switch approach to form gels from different Fmoc-amino acids, whereas Ulijn et al. [13] combined the pH switch method with temperature modulation. Although non-covalent interactions are several times weaker than covalent bonds [14], the presence of multiple intramolecular and intermolecular forces leads to the formation of soft solid-like materials [15]. The physical interactions are dynamic and reversible, an aspect that offers the material the potential to respond to different stimuli [16]. Nevertheless, the reversible nature of non-covalent interactions provides self-healing ability and lacks structural stability. Hence, in situ mixtures of two LMWGs offer the opportunity to enhance their mechanical properties.

Different studies have shown that depending on the selection of LMWGs, depending on the preparation method, the hydrophobicity of the compounds or the complementary functional groups and external environmental triggers, co-assembled systems can lead to orthogonal, disruptive, or cooperative co-assembly [17,18,19]. Either random or specific association between two LMWGs, the mechanism has not been fully elucidated. 

In the present study, we investigated the fundamental principles of self-assembly behavior of five Fmoc-based compounds ((Fmoc-Lys(Fmoc)-OH, (Fmoc-Ser-OH), (Fmoc-Glu), (Fmoc-Gly-Gly-Gly-OH), (Fmoc-Trp-OH) into secondary and higher-order structures using a solvent mixture approach and a pH switch as an external trigger. The presence of Fmoc at both Nα- and Nε-positions of L-lysine provides additional fluorenyl π-π stacking, while the newly provided -CO-NH- linkage facilitates hydrogen bond formation. Furthermore, the methoxycarbonyl group is a potential H-bond acceptor that provides steric optimization. The chemical versatility of Fmoc-Lys(Fmoc)-OH could lead to hydrophobic interactions through the alkyl chain and ionic interactions due to -COOH. Hence, Fmoc-Lys(Fmoc) was chosen as a co-partner for all the aforementioned Fmoc-based compounds.

Furthermore, we investigated the co-assembly pathway of four co-assembled systems: S_1_ based on Fmoc-Lys(Fmoc)-OH and Fmoc-Ser-OH, S_2_ based on Fmoc-Lys(Fmoc)-OH and Fmoc-Glu, S_3_ based on Fmoc-Lys(Fmoc)-OH and Fmoc-Gly-Gly-Gly-OH, S_4_ based on Fmoc-Trp-OH and Fmoc-Lys(Fmoc)-OH. Determining the forces responsible for the co-assembly of co-partners and understanding the role they play in the self-sorting of supramolecular building blocks are essential for predictively designing the structural properties, anisotropy, stability, and bioactivity of the final hydrogel. The self/co-assembly process can occur as a result of the synergistic effect of several types of non-covalent interactions.

Considering the complexity of self- and co-assembly processes, it is necessary to determine the involvement of multiple factors at all length scales. Therefore, the self- and co-assembled systems were analyzed using powder X-ray diffraction (XRD), scanning transmission electron microscopy (STEM), atomic force microscopy (AFM), and polarized optical microscopy (POM) to reveal the molecular-level assembly. For the detection of N-terminal amino group it was used a multi-step procedure, such as the ninhydrin staining assay. 

## 2. Results and Discussion

The study aimed to understand the mechanism of formation of a two-component supramolecular gel using amino acid/peptide molecules as LMWGs. The side chain of amino acids includes a chemical variety from charged, polar, hydrophilic, and hydrophobic groups, which offer a tremendous diversity of non-covalent intermolecular interactions leading to the formation of supramolecular structures.

Table 1 presents the structures of Fmoc-amino acids and Fmoc-tripeptide used in this study as LMWGs, whereas Table 2 presents the two-component mixtures formed by the co-assembly of Fmoc-Lys(Fmoc)-OH (M_1_) and an amino acid (Fmoc-Ser-OH (M_2_), Fmoc-Glu (M_3_), Fmoc-Trp-OH (M_5_) or a short peptide (Fmoc-Gly-Gly-Gly-OH (M_4_)) as co-partner. According to Table 2, the S_1_, S_2_, and S_3_ co-assembled systems were formed by mixing M_1_:co-partener (M_2_/M_3_/M_4_) in a 5:1 gravimetric ratio, whereas S_4_ was formed by mixing M_1_ and M_5_ in a 1:1 gravimetric ratio. The aforementioned co-assembled systems have been extensively characterized and discussed from a physicochemical point of view in previously reported work [20,21].

According to Table 2, only the M_1_ and M_5_ precursors form self-assembled gels as single-component systems, whereas M_2_, M_3_, and M_4_ form transparent viscous solutions. In the case of co-assembled gels, the gelation process was easily detected by simply using the vial inversion method. As can be seen from Table 2, the S_1_, S_2_ and S_4_ obtained gels presented transparent, while S_3_ gel had a translucent appearance.

### 2.1. Detection of N-Terminal Amino Groups through Ninhydrin Assay

The presence of the Fmoc group provides double functionality, as a protecting group in peptide synthesis and as an enhancer in the self-assembly process via aromatic stacking interactions. The Fmoc compounds used in this study formed hydrogels in a polar aprotic solvent (DMSO) and phosphate-buffered solution mixture. Literature data have shown that DMSO can lead to Fmoc cleavage under specific conditions [22]. Hence, the ninhydrin (2,2-dihydroxyindane-1,3-dione) assay, an alternative to the Kaiser test [23], was used to detect N-terminal amino groups. The principle of this colorimetric assay is based on the formation of a purple–blue complex (‘Ruhemann’s purple’) formation when ninhydrin detects the α-amino group of primary amines [24,25]. 

**Figure 1:**
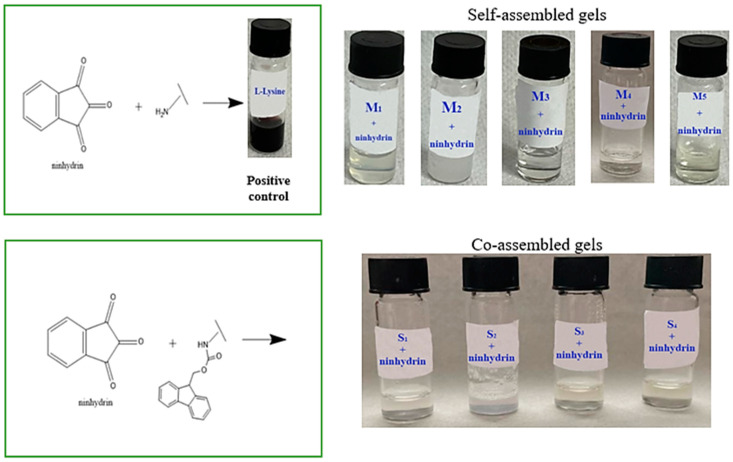
Staining by ninhydrin solution for detection of N-terminal amino groups.

Figure 1 presents the ninhydrin assay results. L-lysine with free functional groups was used as a positive control. When the ninhydrin solution was added to the L-lysine solution, a purple color was observed because the free primary amine reacted with it. According to Konno et al. [23], if no color change is observed, it indicates the absence of a free amine group. Figure 1 presents the ninhydrin assay results. L-lysine with free functional groups was used as a positive control. When the ninhydrin solution was added to the L-lysine solution, a purple color was observed because the free primary amine reacted with it. 

The self- and co-assembled gels did not show any color change due to the presence of the Fmoc group at the N-terminal. These results suggest that DMSO does not lead to Fmoc-deprotection.

### 2.2. X-ray Powder Diffraction (XRD)

The crystalline, semicrystalline and amorphous nature of the self- and co-assembled systems was analyzed by powder XRD technique. Diffraction patterns provide the necessary information to identify crystalline, semicrystalline, and amorphous materials. XRD diffractograms were collected for the lyophilized form of the samples and provided insight into the packing of amino acids/short peptide molecules. 

**Figure 2:**
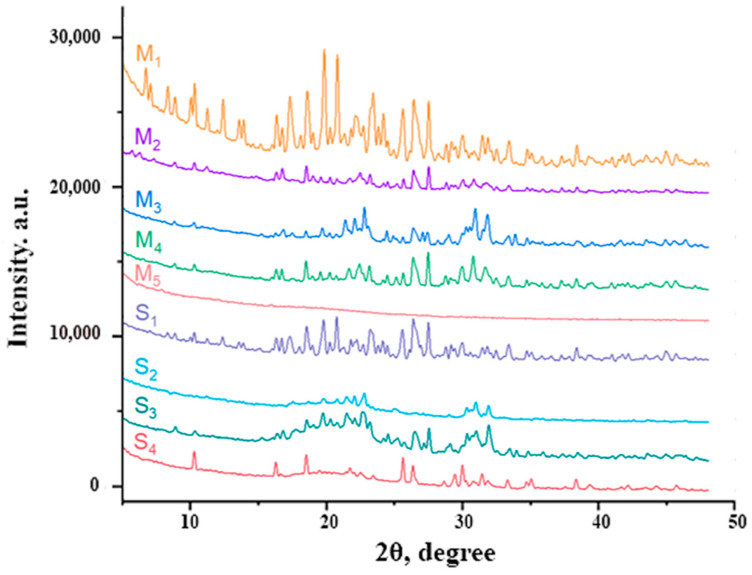
Powder X-ray diffraction of precursor components (M_1_–M_5_) and co-assembled gels (S_1_–S_4_).

Figure 2 show the XRD pattern of the co-assembled systems and their precursor components, while in Table 3 are presented the significant XRD peaks (2θ) and corresponding interplanar d-spacing. As can be seen, the main peaks which appear in the XRD diffractogram of precursor compounds are 2θ = ~10°, 2θ = ~17°, 2θ = ~19° and 2θ = ~23°. The XRD diffraction pattern for co-assembled systems contained distinct peaks at 2θ = ~9°, 2θ = ~17° and 2θ = ~23°.

The interplanar d-spacing was determined using Bragg’s Law. As expected, the peaks of d-spacing at 3.8 Å and 3.4 Å appeared in either self- or co-assembled systems (except M_5_) due to the π-π stacking between aromatic rings generated by fluorenyl rings of Fmoc groups [26,27]. 

The XRD diffractogram of the M_1_ self-assembled system shows a broad zone of different peak intensities with their d-spacing values between 2–6 Å associated with an amorphous phase [28]. Furthermore, the peaks of d-spacing at 4.9 Å and 10.54 Å are attributed to the inter-sheet distance or to antiparallel β-strands spacing [29]. The presence of a peak at 4.2 Å is attributed to π-π stacking. The reflection peaks at 4.6 Å and 5.1 Å which appear in the XRD diffractogram of the M_2_ gelator correspond to hydrogen bonding between β-strands [29]. The XRD diffractograms of M_3_ and M_4_ show similar packing with slight differences. The peak of d-spacing at 8.3 Å is assigned to the α-helix structure [30]. A distinct peak of d-spacing at 9.7 Å is observed in the XRD pattern of M_4_. A noisy pattern without peaks is seen in the case of the M_5_ sample, suggesting an amorphous material.

The XRD diffractograms of the co-assembled systems present the characteristic peaks of d-spacing at 3.8 Å and 3.4 Å. It is also evident that co-assembled semicrystalline materials are generated by the interaction of the two precursor compounds.

Table 3 presents the significant peaks obtained via XRD and corresponding interplanar d-spacing of both precursor components (M_1_–M_5_) and co-assembled systems (S_1_–S_4_).

### 2.3. Congo Red Staining and Polarized Optical Microscopy

**Figure 3:**
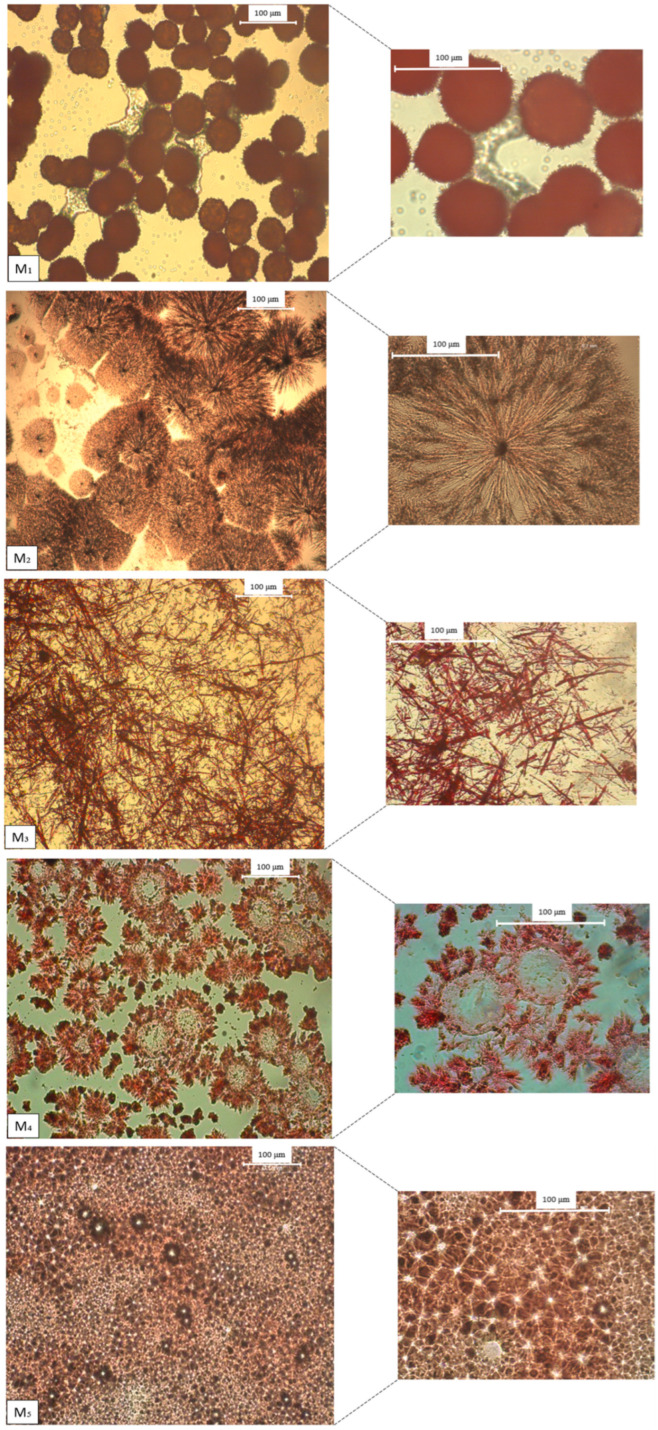
Polarized optical microscopy images of self-assembled Fmoc-AA stained with Congo red. The scale bar represents 100 µm.

In recent years, various studies have focused on the development of self-assembled materials. However, the formation of nanoscale assemblies has not been fully elucidated. Therefore, a Congo red staining assay was used to investigate the presence of amyloid fibers in self-assembled/co-assembled systems based on Fmoc-modified amino acids [31,32]. The characteristic birefringence of the amyloid fibers was monitored using polarized optical microscopy (POM). POM images of self-assembled Fmoc-AA stained with Congo red showed different nanoaggregate morphologies (Figure 3).

As can be seen from Figure 3, the M_1_ gelator forms a dendrimer-like self-assembled structure. According to the study realized by Stagi et al. [33], L-lysine is an AB_2_-type amino acid that can form a branched structure via its functional groups such as carboxylic acids and amines [34]. Taking into account that the amino groups in the α and ε positions form -CO-NH- bonds with Fmoc groups, we can conclude that M_1_ adopts a dendrimer-like structure due to hydrogen bonds [35,36]. The POM image of the M_2_ gelator stained with Congo red shows a highly ordered self-assembled spherulite-like structure with a birefringence texture characteristic of amyloid fibrils. The hydrophilic residue of Fmoc-serine tends to form hydrogen bonds, whereas the molecular packing of M_3_ may be influenced by the presence of the two carboxylic groups [37]. In the presence of Congo red, M_3_ reveals a fibrillar self-assembled structure. In the case of the M_4_ gelator, upon Congo red staining, the molecules exhibited strong birefringence and a vesicle-like self-assembled structure, while M_5_ forms a dense fibrillar network as shown in Figure 3. 

**Figure 4:**
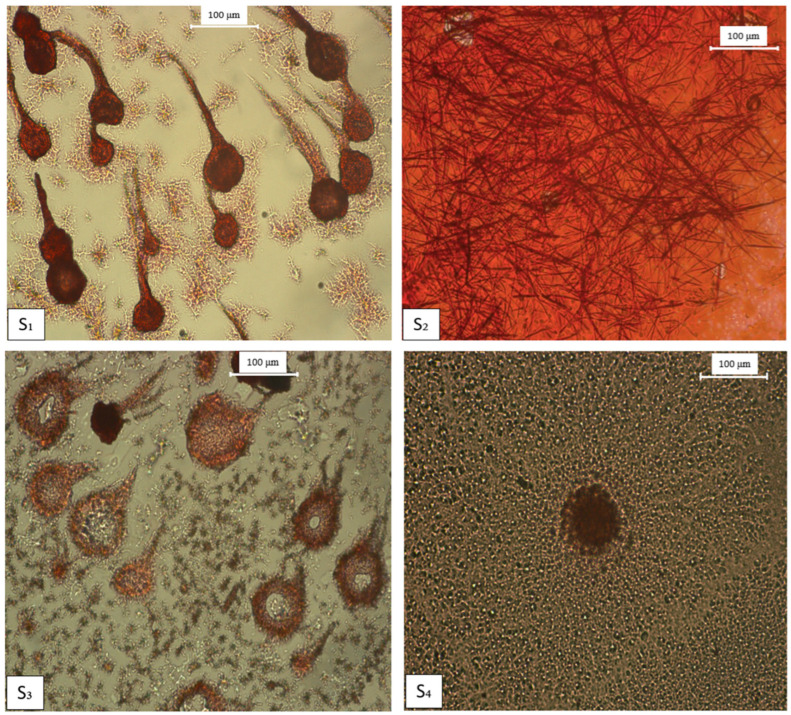
Polarized optical microscopy images of co-assembled systems stained with Congo red. The scale bar represents 100 µm.

Figure 4 presents the polarized optical microscopy images of the co-assembled systems stained with Congo red.

The co-assembled systems present different morphologies than the initial systems, which indicate the interaction between the compounds. Therefore, the POM image of the S_1_ system shows both the dendrimer-like structure characteristic of M_1_ and the fibrils characteristic of M_2_. This behavior is also observed for the S_2_, S_3_, and S_4_ co-assembled systems.

### 2.4. Atomic Force Microscopy

**Figure 5:**
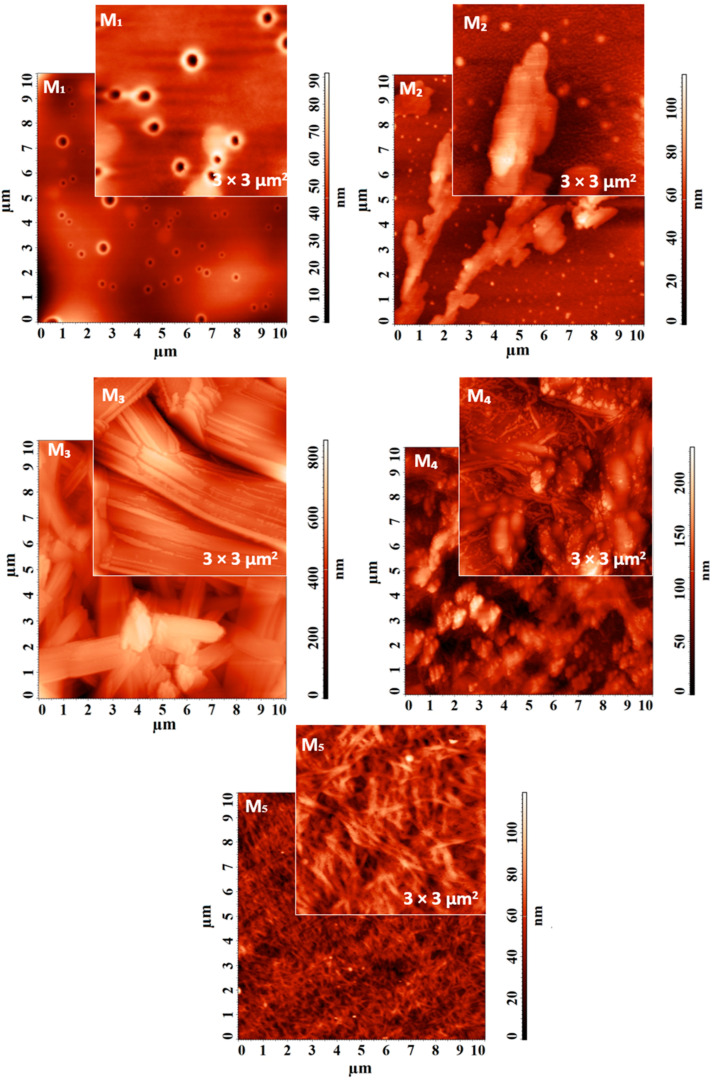
AFM topographical images of self-assembled Fmoc-AA (M_1_, M_2_, M_3_, M_4_, M_5_) collected on 10 × 10 µm^2^ and 3 × 3 µm^2^.

**Figure 6:**
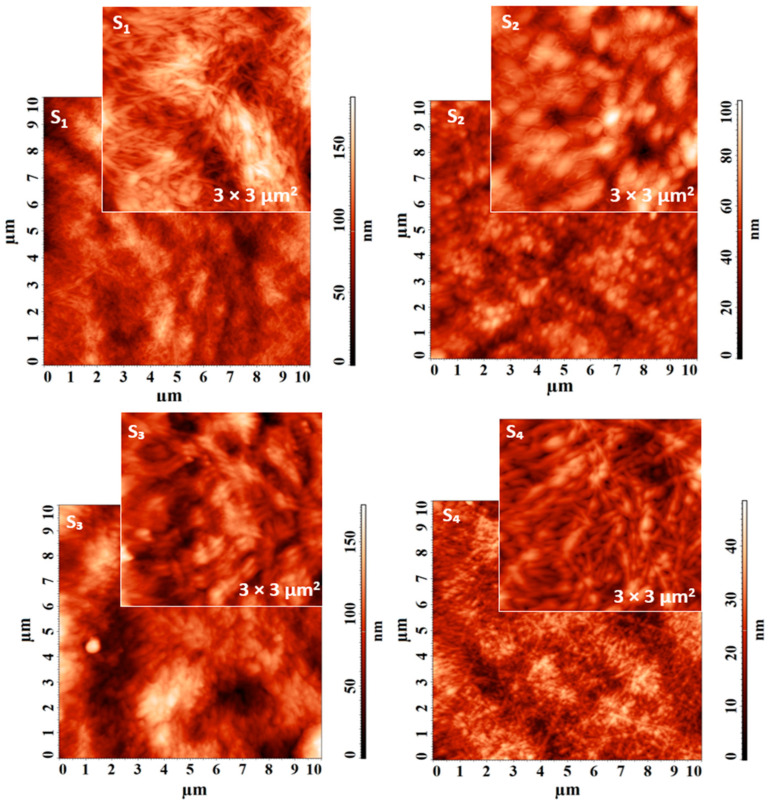
AFM topographical images of co-assembled systems (S_1_, S_2_, S_3_, S_4_) collected on 10 × 10 µm^2^ and 3 × 3 µm^2^.

AFM was used to investigate the self-assembled Fmoc-AA and co-assembled systems at the nanoscale, by the quality of the images collected on different scan sizes and presented Figure 5 for M_1_, M_2_, M_3_, M_4_, and M_5_ self-assembled samples and in Figure 6 for S_1_, S_2_, S_3_, S4 co-assembled samples. As an overview, it can be observed that the surface morphology is strongly influenced by the structure of the analyzed supramolecular material. All the average dimensions of the structures were calculated using the statistical analysis of the cross-sectional profiles taken on each surface feature.

Thus, M_1_ presents vesicle-like self-assembled structures with a mean inner diameter of 129 ± 24 nm and mean external diameter of 318 ± 57 nm, evenly distributed over the entire analyzed surface, as observed in Figure 5. Although some of these fibers seem to be oriented towards a preferential direction, overall, a morphologically isotropic structure is still created. The self-assembled M_2_ structure presents fern-like dendritic spherulite morphologies on wide scanning areas. Details of the branches with larger widths of about 815 ± 120 nm are visible in the AFM images from Figure 5. M_3_ sample reveals large width randomly distributed fibers, having an average diameter of 490 ± 140 nm. The detailed AFM image suggests that molecules of M_3_ are organized in fibers whereupon they actually interact and form a ribbon-like structure composed of multiple overlapping layers, with a slight tendency to break. The topographical images obtained for the self-assembled M_4_ system and displayed in Figure 5 reveal two types of morphological entities on the surface, which are interspersed. The first is represented as a raw material, from which the second type of entity, made up of fibers with an average diameter of 119 ± 41 nm, is formed. In contrast to the previous cases, the AFM images reveal the formation of a nanostructured thick fibrillar network with fibers with an average diameter of 72 ± 9 nm for M_5._

For all co-assembled systems, it was demonstrated once again the formation of fibers, with different morphologies, according to the chemical structure of each system, as seen in Figure 6, especially on the small scanning area of 3 × 3 µm^2^. In the case of the S_1_ sample, the fibers were well defined, slightly strangled in some areas, forming a fairly dense and packed network, as seen in Figure 6. Their mean diameter was 67 ± 13 nm. On the other hand, the S_2_ sample presents fibrillar formations arranged more widely on a granular substrate, being much narrower, having an average width of 40 ± 6 nm, as observed in the detailed AFM image from Figure 6. S_3_ contains the thickest fibers of the four co-assembled systems, with an average diameter of 114 ± 15 nm and an apparent parallel arrangement at the studied scanning level. This aspect can be noticed in Figure 6. The S_4_ system was co-assembled into fibers with a mean size of 85 ± 17 nm that create globular formations in some spots during their path, with an average diameter of 174 ± 36 nm. Both AFM topographical images collected on large and reduced scanning areas highlight this. 

### 2.5. STEM

Scanning transmission electron microscopy (STEM) was used to provide insight into the morphology of both precursor compounds and co-assembled systems [38]. 

**Figure 7:**
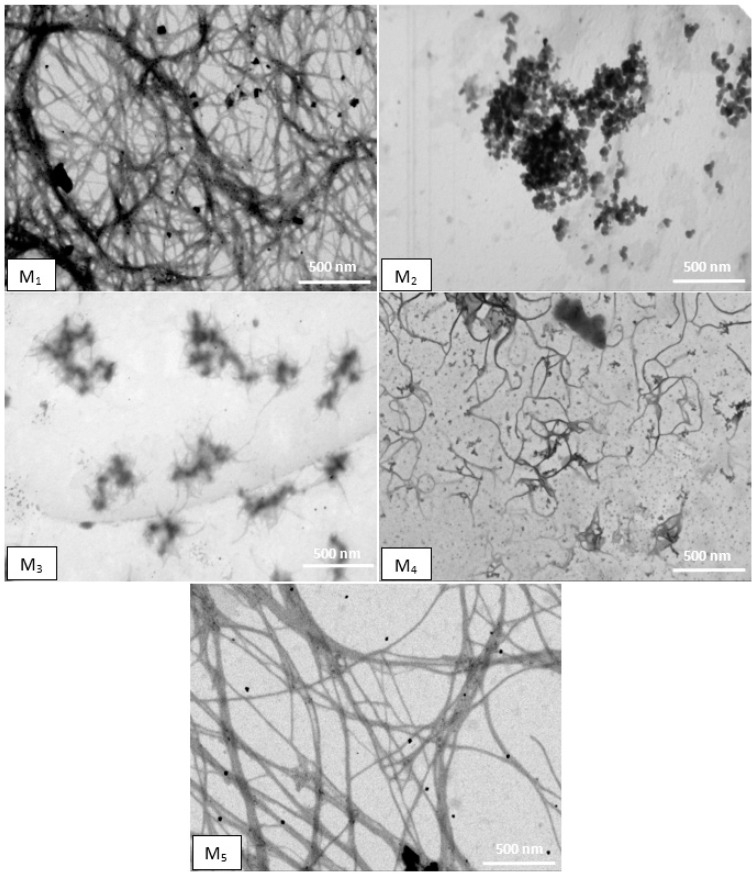
STEM images of precursor compounds. The scale bar represents 500 nm.

As shown in Figure 7, the molecules of precursor compounds were organized in different morphologies after gelation. The STEM image of M_1_ presents a dense fibrillar network, while POM and AFM morphologies revealed a dendrimer-like structure. Following this aspect, it can be concluded that the Fmoc-Lys(Fmoc)-OH molecules initially associate in a dendrimer-like structure, after which they interact and unite, finally forming a fibrous network. In the case of M_2_ molecules, they self-assemble in solution forming aggregates in the form of spherulites. The aforementioned results along with the STEM results show that molecules of M_3_ are initially organized in fibers whereupon they overlap. Shorter and more flexible fibers are observed in the STEM image of M_4_, while M_5_ presents a fibrous network with widespread branching points.

**Figure 8:**
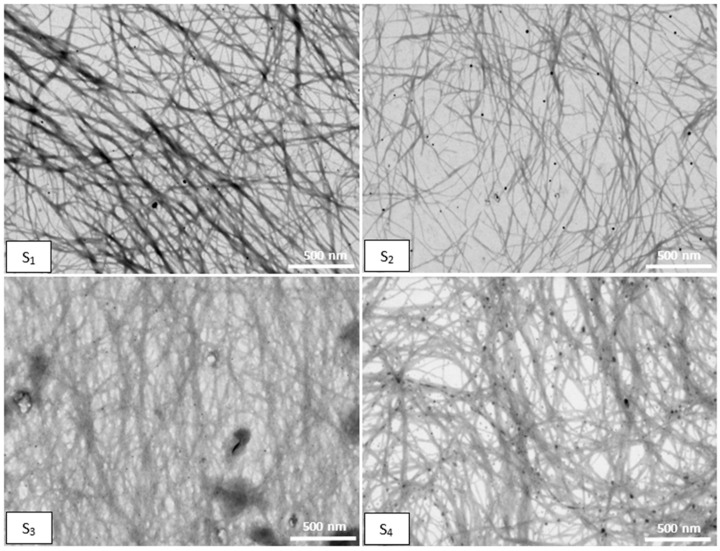
STEM images of co-assembled systems. The scale bar represents 500 nm.

Figure 8 confirms the presence of fibers forming a dense fibrillar network in all the co-assembled systems. However, some differences appear between the systems. The S_1_ system is composed of straight nanofibers aligned side-by-side, while S_2_ presents a less dense network with shorter and more ductile nanofibers. The STEM images of S_3_ and S_4_ systems show a dense entangled network with some branching points.

## 3. Conclusions

In the study, it was demonstrated that the aggregation of LMWG-type molecules, followed by the capture of large amounts of water/solvent, gave rise to four 3D networks. Using the solvent/co-solvent technique, three co-assembled supramolecular systems (S_1_, S_2_ and S_3_) were obtained, while the pH changing led to a co-assembled supramolecular system (S_4_).

XRD results, along with STEM, AFM, and POM microscopy show that all co-assembled gels possess fibrous morphology with slight differences. These differences are generated by the side groups of the amino acid, the length of the tripeptide, the trigger method approach and the interactions that occur between the molecules. Hence, it can be concluded that the initial compounds have different morphologies, but through co-assembly they form fibrous networks. Furthermore, the Fmoc motif present at the N-terminal of amino acids/tripeptide facilitates gelation through additional π-π interactions, while the methoxy linker is a potential hydrogen bond acceptor providing steric optimization.

## 4. Materials and Methods

### 4.1. Materials

The Fmoc-Lys(Fmoc)-OH amino acid (C_36_H_34_N_2_O_6_, Mw = 590.66 g/mol) was purchased from Sigma-Aldrich (Darmstadt, Germany). The Fmoc-Ser-OH (C_18_H_17_NO_5_, Mw = 327.34 g/mol) and Fmoc-Glu (C_20_H_19_NO_6_, Mw = 369.37 g/mol) amino acids were acquired from Alfa Aesar (Kandle, Germany), while Fmoc-Trp-OH (C_26_H_22_N_2_O_4_, Mw = 426.46 g/mol) was provided by Novabiochem (Darmstadt, Germany). The Fmoc-Gly-Gly-Gly-OH (C_21_H_21_N_3_O_6_, Mw = 411.41 g/mol) tripeptide was purchased from Bachem (Bubendorf, Switzerland). All compounds presented a standard 9-fluorenyl methoxycarbonyl (Fmoc) group and were used as received without further purification.

As solvents were used dimethyl sulfoxide (DMSO) from Fluka (Buchs, Switzerland), sodium hydroxide (NaOH) and citric acid from Sigma-Aldrich (Darmstadt, Germany). Sodium phosphate buffer (PBS, pH 7.4, 0.01 M) was prepared via standard protocol. Congo Red and ninhydrin were purchased from ABCR (Karlsruhe, Germany).

### 4.2. Methods

The formation of the supramolecular gels was realized by individually preparing of the precursor solutions and by mixing them in 5:1 gravimetric ratio, respectively 1:1 gravimetric ratio. The S_1_, S_2_ and S_3_ gels were prepared using a polar solvent, whereas the S_4_ supramolecular gel was prepared using a pH change technique. 

Each precursor compound (Fmoc-Lys(Fmoc)-OH (M_1_) and Fmoc-Ser-OH (M_2_) in the case of S_1_ gel, Fmoc-Lys(Fmoc)-OH (M_1_) and Fmoc-Glu (M_3_) for S_2_ gel and Fmoc-Lys(Fmoc)-OH (M_1_) and Fmoc-Gly-Gly-Gly-OH (M_4_) for S_3_ supramolecular gel) was dissolved in DMSO. When the solution became transparent 0.01 M phosphate buffer (pH = 7.4) was gradually added. The ratio between phosphate-buffered solution and solvent was 60:1. For the preparation of the S_4_ gel, Fmoc-Lys(Fmoc)-OH (M_1_) was prepared as mentioned above, while Fmoc-Trp-OH (M_5_) solution was prepared by dissolving the powder in 0.05 M NaOH. After complete dissolution, the pH was changed using a few drop-wise of 0.1 M citric acid (~20 µL). As a last step, 0.01 M phosphate-buffered solution (pH = 7.4) was added and the solution was gently stirred until the mixture became translucent.

The self-assembled gels were prepared in accordance with aforementioned excepting the step in which the two precursor compounds are mixed.

### 4.3. Detection of N-Terminal Amino Groups through Ninhydrin Assay

Detection of N-terminal amino groups was realized through the conventional ninhydrin assay [39] with slight modifications. Then, 2% *w*/*v* ninhydrin solution in EtOH was used to stain primary amines. L-lysine was used as a positive control, whilst Fmoc-Lys(Fmoc)-OH (M_1_), Fmoc-Ser (M_2_), Fmoc-Glu (M_3_), Fmoc-Gly-Gly-Gly (M_4_), Fmoc-Trp-OH (M_5_), and co-assembled systems (S_1_, S_2_, S_3_, S_4_) were analyzed samples. The self- and co-assembled systems were tested as follows: 1 mL of sample solution was mixed with 50 µL of freshly prepared ninhydrin. The mixture was heated for 2 min at 95 °C in a boiling water bath. The free or protected α-amino groups were revealed depending on the color development.

### 4.4. X-ray Powder Diffraction (XRD)

Powder diffraction analyses were carried out on a Shimadzu XRD-6000 diffractometer with Cu-Kα radiation (ƛ = 1.5406). Samples were scanned in the angular range of 2–50° (2θ) with 0.01° step and a recording rate of 2°/min. The d-spacing was calculated using Bragg’s Law:

$$d-spacing=(\frac{ƛ}{2sin\theta })$$

where ƛ is the X-ray wavelength and θ is the Bragg’s angle in radians.

### 4.5. Polarized Optical Microscopy (POM)

The supramolecular architectures of the self-assembled Fmoc-AA (M_1_, M_2_, M_3_, M_4_, M_5_) and co-assembled systems (S_1_, S_2_, S_3_, S_4_) were studied by using POM images which were obtained using a Leica DM2500 M microscope (Leica Microsystems, Wetzlar, Germany).

### 4.6. Congo Red Staining Analysis

For the preparation of Congo red solution, a sodium chloride/EtOH saturated solution was initially prepared by adding sodium chloride in excess in the 8:2 EtOH–water mixture. The resulting mixture was filtered after 10 min of stirring. After that, Congo red was added in excess to sodium chloride/EtOH solution, stirred and filtered after 10 min.

The self- and co-assembled gels were prepared as we reported in our previous studies [16,17]. Subsequently, 50µL of the sample was dried on a microscope slide for 24 h and stained with 10 µL of fresh Congo red solution. After 2 min, Congo red was removed by blotting and the stained sample was covered with a microscope coverslip. 

### 4.7. Atomic Force Microscopy (AFM)

AFM topographical images of self-assembled Fmoc-AA (M_1_, M_2_, M_3_, M_4_, M_5_) and co-assembled systems (S_1_, S_2_, S_3_, S_4_) were registered on 10 × 10 µm^2^ and 3 × 3 µm^2^ scanning areas, using a NTEGRA Scanning Probe Microscope (NTMDT, Zelenograd, Moscow, Russia). The measurements were performed at room temperature in tapping mode. The resonance frequency of the NSG03 cantilever (TipsNano Co., Tallinn, Estonia) was 82 kHz. The Nova software 1.0.26.1443 from NT-DMT was used to obtain and analyze the AFM images. 

### 4.8. STEM

STEM microscopy was carried out on Verios G4 UC (Thermo Scientific, BRNO, Czech Republic) working in STEM mode at 15 kV, with a STEM 3+ detector (Bright-Field Mode). After 24 h of gelling, the samples were deposited on a carbon-coated copper grid (300 mesh) and were left overnight in an oven at 30 °C. 

## Figures and Tables

**Table 1:** Structures of Fmoc-amino acids and Fmoc-tripeptide used in this study as LMWGs.

Sample Code	Name	Structure
M_1_	Fmoc-Lys(Fmoc)-OH	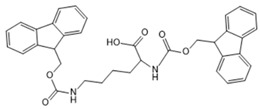
M_2_	Fmoc-Ser-OH	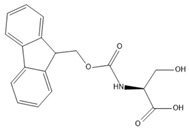
M_3_	Fmoc-Glu	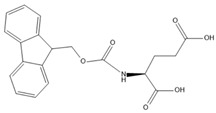
M_4_	Fmoc-Gly-Gly-Gly-OH	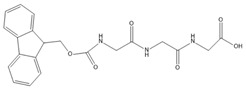
M_5_	Fmoc-Trp-OH	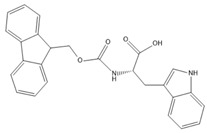

**Table 2:** Appearance of S_1_–S_4_ co-assembled gels obtained from M_1_–M_5_ as precursor components.

Gelator	Trigger	Appearance of Self-Assembled Gel at pH 7.4	
M_1_	Solvent/phosphate-buffered solution	Transparent gel	
M_2_	Solvent/phosphate-buffered solution	Transparent solution	
M_3_	Solvent/phosphate-buffered solution	Transparent solution	
M_4_	Solvent/phosphate-buffered solution	Transparent solution	
M_5_	pH switch	Slightly turbid gel	
Systems	Co-parteners	Ratio	Appearance of co-assembled gel at pH 7.4
S_1_	M_1_:M_2_	5:1	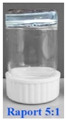
S_2_	M_1_:M_3_	5:1	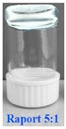
S_3_	M_1_:M_4_	5:1	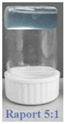
S_4_	M_1_:M_5_	1:1	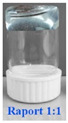

**Table 3:** Significant XRD peaks and corresponding interplanar d-spacing.

Sample	2θ (°)	d-Spacing (Å)	Sample	2θ (°)	d-Spacing (Å)
M_1_	8.38	10.54	S_1_	9.11	9.7
	17.79	4.9		23.34	3.8
	21.16	4.2			
M_2_	17.25	5.1	S_2_	23.21	3.8
	19.11	4.6			
	23.23	3.8			
M_3_	10.54	8.39	S_3_	8.89	9.9
	19.13	4.6		17.76	4.9
	23.40	3.8		23.40	3.8
M_4_	9.03	9.7	S_4_	16.77	5.2
	10.60	8.34			
	19.27	4.6			
	23.26	3.8		19.07	4.6
M_5_	no peaks				
				26.13	3.4
				27.36	3.2

## Data Availability

The data presented in this study are available on request from the corresponding author. The data are not publicly available due to privacy and ethical restriction.
